# Supplementary material for: Brain Delivery of IGF1R5, a Single-Domain Antibody Targeting Insulin-like Growth Factor-1 Receptor
Source: Pharmaceutics. 2022 Jul 12;14(7):1452. doi: 10.3390/pharmaceutics14071452 (PMC9316817; doi:10.3390/pharmaceutics14071452)
Supplement: Supplementary file 1 [file pharmaceutics-14-01452-s001.zip › pharmaceutics-1796494-supplementary.pdf]

```
                                                                                                                   1
                     1         2         3         4         5          6         7         8            9         0                1
(Kabat)     1234567890123456789012345678901234567890123456789012 3A45678901234567890123456789012ABC345678901234567890ABCDEFG1234567890123

IGF1R5      QVKLEESGGGLVQAGGSLRLSCAASGRTIDNYAMAWSRQAPGKDREFVATIDWGDGGARYANSVKGRFTISRDNAKGTMYLQMNNLEPEDTAVYSCAMARQSRVNLDVARYDYWGQGTQVTVSS
IGF1R5-H2   QVQLVESGGGLVQPGGSLRLSCAASGRTIDNYAMAWVRQAPGKGLEWVATIDWGDGGTRYANSVKGRFTISRDNSKNTMYLQMNSLRAEDTAVYYCAMARQSRVNLDVARYDYWGQGTLVTVSS
            *                                               *                             *                  *
```

Supplemental Figure S1. Sequence alignment between the camelid IGF1R5 sdAb and the humanized variant IGF1R5-H2. Kabat numbering scheme is shown on top. CDRs are shown in red and underlined. FR mutations introduced in IGF1R5-H2 are shown in white on black background. Back-mutations introduced in the human FR template are highlighted by asterisks. A mutation in CDR2 required for Protein A binding is shown in red on black background.

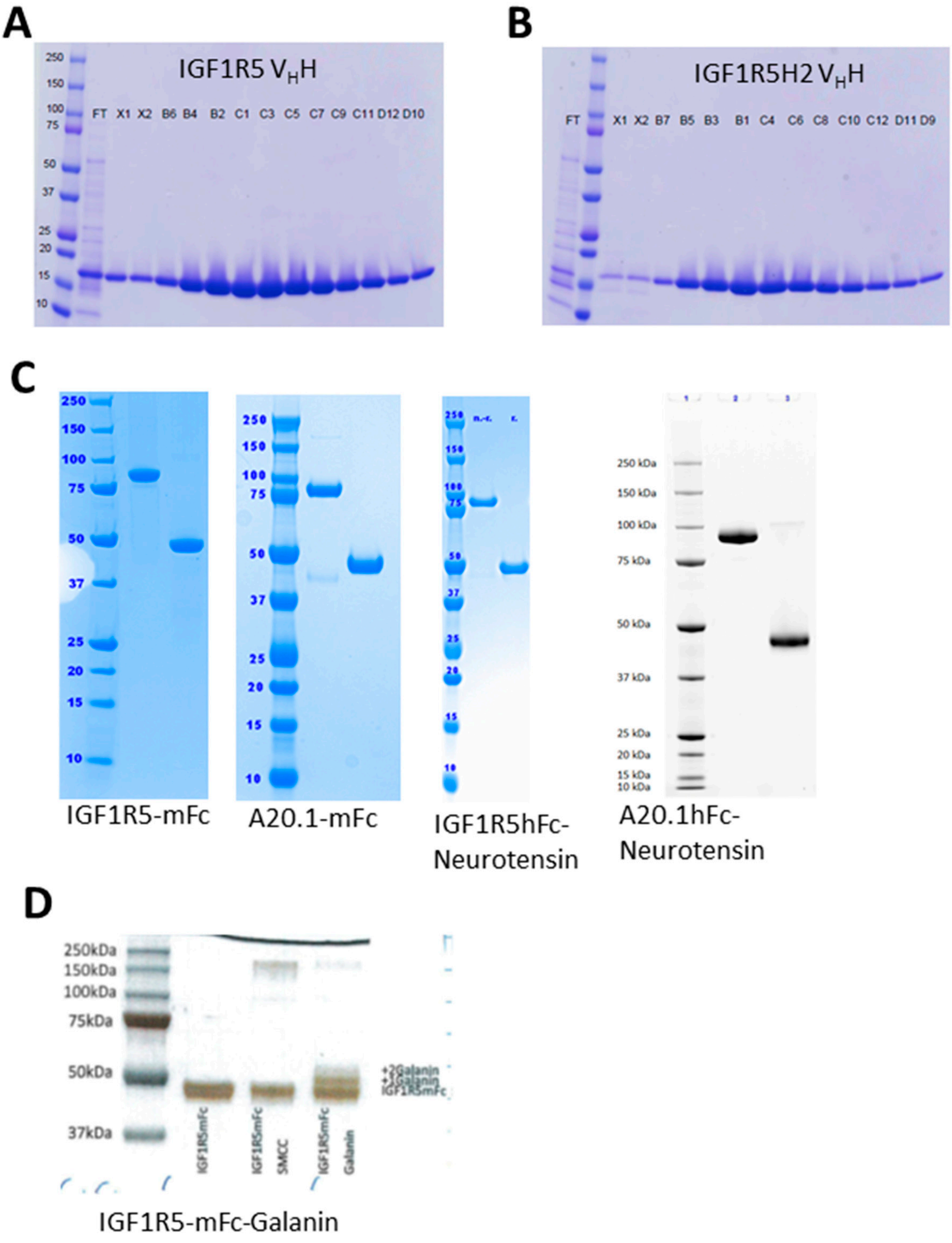

Supplemental Figure S2: Representative SDS-PAGE images of IGF1R5 constructs. (A,B) Reducing SDS-PAGE was used to analyze the purity of the IGF1R5 VHH (A) and IGF1R5-H2 VHH (B) following His-TRAP purification. 10 μL of sample was loaded in each lane. (C) SDS-PAGE profile of purified IGF1R5-mFc, A20.1-mFc, IGF1R5hFc-Neurotensin and A20.1hFc-Neurotensin under reducing and non-reducing conditions. (D) Molecular weight shift IGF1R5mFc-galanin versus IGF1R5mFc and IGF1R5mFc-SMCC in a silver stained SDS-PAGE gel under reducing conditions.

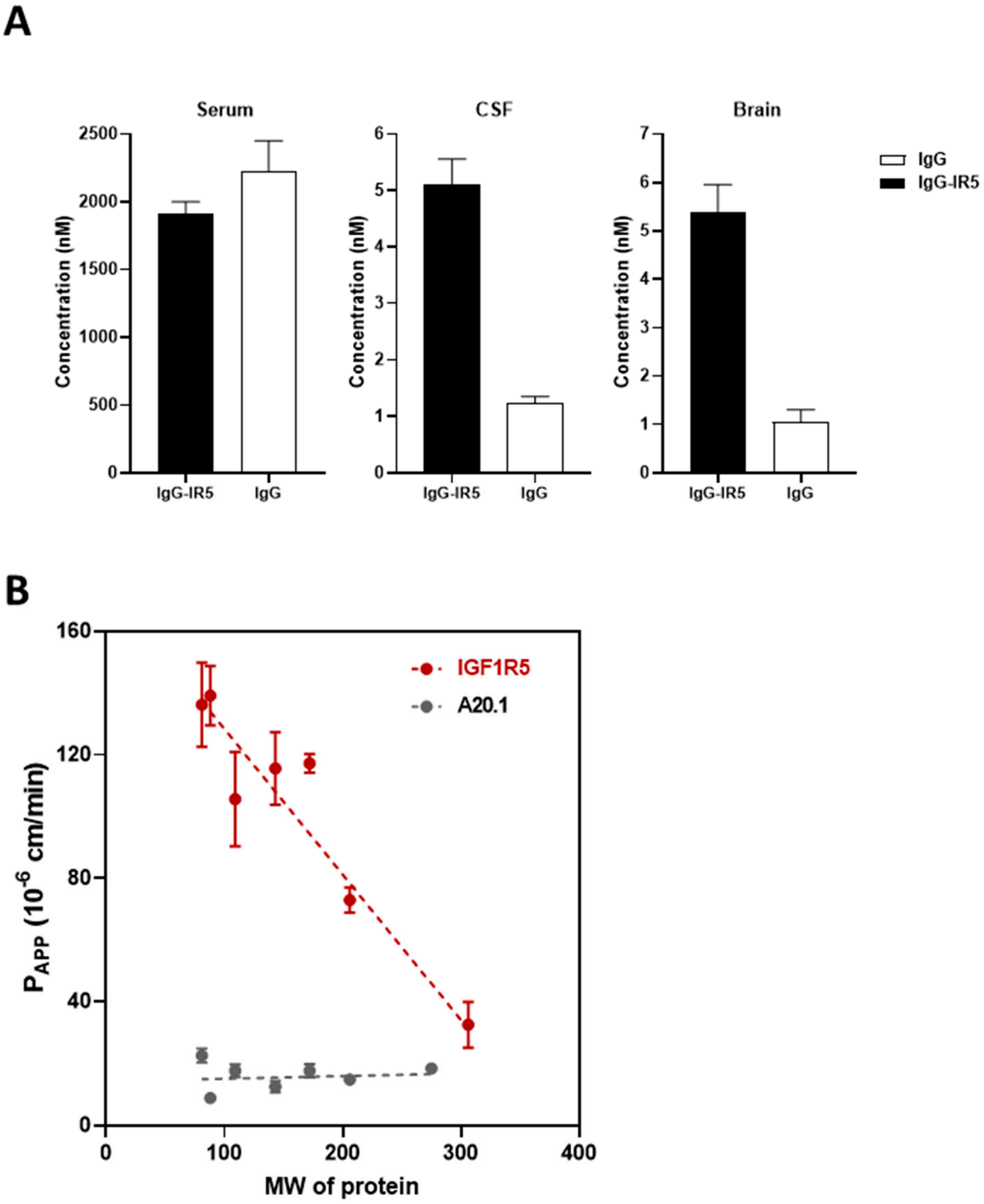

Supplemental Figure S3. Rat (SV-ARBEC) in vitro BBB transport of IgG-fused IGF1R5 and molecular weight/transmigration value of various IGF1R5 contructs. (A) Concentrations of a non-BBB crossing IgG either not fused (grey bars) or fused with IGF1R5 (red bars) in serum, CSF and brain of rat at 24 h following a bolus i.v. injection of 30 mg/kg of each antibody. The concentrations were measured using SRM analysis in at least 5 animals and bars represent mean and SD. (B) IGF1R5 and A20.1 constructs with variable molecular weights were added to the upper compartment of the BBB insert and then quantified over time in the bottom chamber using SRM to determine Papp values. Papp values (cm/min) of antibodies are shown as means ± sd derived from 6 separate Transwell inserts.

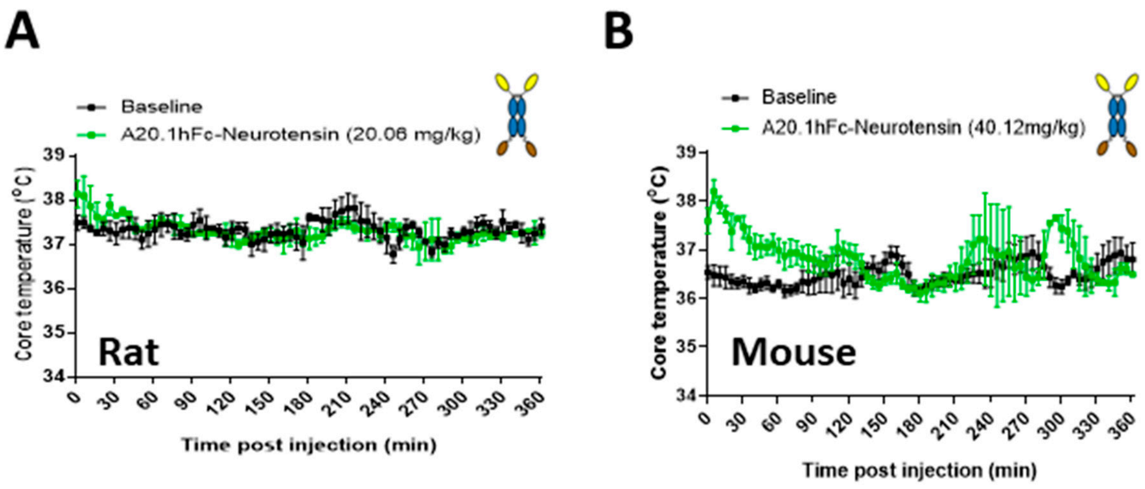

Supplemental Figure S4: A20.1hFc-Neurotensin injection in rats and mice. Effect of an intravenous injection of A20.1hFc-Neurotensin in rats (20.06 mg/kg, n=3) (A) and mice (40.12 mg/kg, n=4) (B). Core body temperature was monitored by telemetry up to 6 h post intravenous injection of test compounds. Results are mean ± SEM of 3-4 animals.

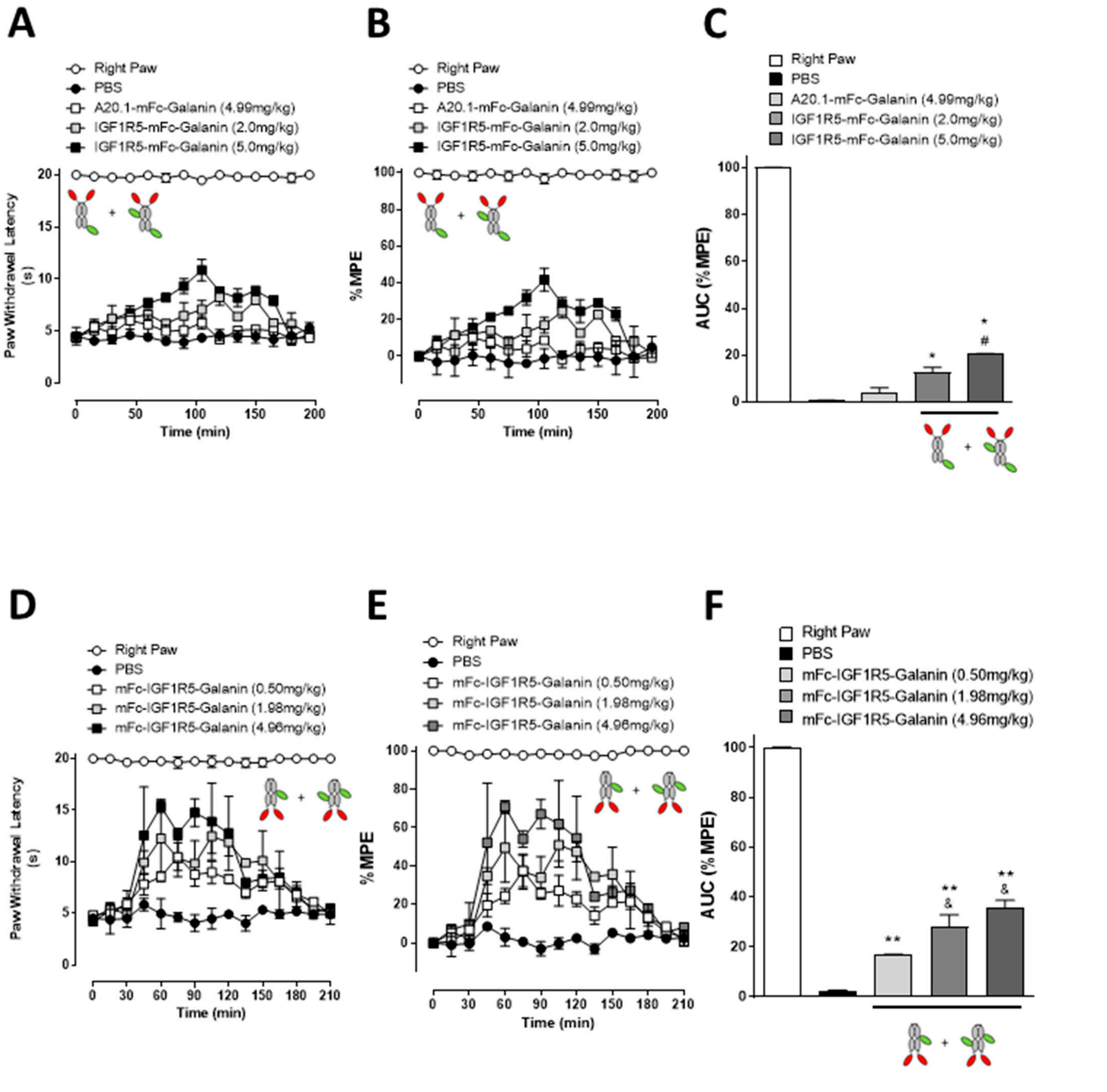

Supplemental Figure S5: Reversal of thermal hyperalgesia induced by IGF1R5-mFc-Galanin and mFc-IGF1R5-Galanin in the Hargreaves model of inflammatory pain. Dose-response of latency paw withdrawal of control right paw or inflamed paw to a thermal stimulus was measured at 15 min interval after intravenous injection of IGF1R5-mFc-Galanin (A) or mFc-IGF1R5-Galanin (D). Responses to each compound are also expressed as percentage of maximal possible effect (MPE) of the control paw (B and E). MPE curves were used to integrate area under the curve (AUC) of the paw withdrawal latency curve (C and F). Data are shown as mean ± SEM of 2-4 rats per group. A20.1-mFc-Galanin (n=3); IGF1R5-mFc-Galanin (2.0mg/kg, n=2); IGF1R5-mFc-Galanin (5.0mg/kg, n=2); mFc-IGF1R5-Galanin (0.50mg/kg, n=2); mFc-IGF1R5-Galanin (1.98mg/kg, n=2); mFc-IGF1R5-Galanin (4.96mg/kg, n=2). *$p<0.01$ vs A20.1-mFc-Galanin; # $p<0.05$ vs IGF1R5-mFc-Galanin (2.0 mg/kg); ** $p<0.01$ vs PBS; & $p<0.05$ vs mFc-IGF1R5-Galanin (0.5 mg/kg).
